# The Relevance, Predictability, and Utility of Annexin A5 for Human Physiopathology

**DOI:** 10.3390/ijms25052865

**Published:** 2024-03-01

**Authors:** Jian Jing

**Affiliations:** Beijing Key Laboratory of Biotechnology and Genetic Engineering, College of Life Sciences, Beijing Normal University, Beijing 100875, China; jjing@bnu.edu.cn; Tel.: +86-010-58802065

**Keywords:** human annexin A5, phosphatidylserine (PS), physiopathology, self-assembly

## Abstract

As an important functional protein molecule in the human body, human annexin A5 (hAnxA5) is widely found in human cells and body fluids. hAnxA5, the smallest type of annexin, performs a variety of biological functions by reversibly and specifically binding phosphatidylserine (PS) in a calcium-dependent manner and plays an important role in many human physiological and pathological processes. The free state hAnxA5 exists in the form of monomers and usually forms a polymer in a specific self-assembly manner when exerting biological activity. This review systematically discusses the current knowledge and understanding of hAnxA5 from three perspectives: physiopathological relevance, diagnostic value, and therapeutic utility. *h*AnxA5 affects the occurrence and development of many physiopathological processes. Moreover, hAnxA5 can be used independently or in combination as a biomarker of physiopathological phenomena for the diagnosis of certain diseases. Importantly, based on the properties of hAnxA5, many novel drug candidates have been designed and prepared for application in actual medical practice. However, there are also some gaps and shortcomings in *h*AnxA5 research. This in-depth study will not only expand the understanding of structural and functional relationships but also promote the application of hAnxA5 in the field of biomedicine.

## 1. Introduction

**Figure 1:**
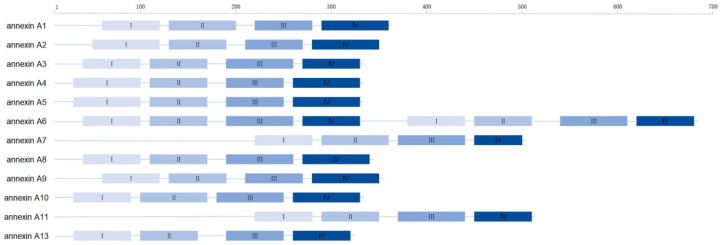
Schematic representation of the structures of all human annexin class A proteins.

Annexins are a family of water-soluble and cytosolic proteins that bind to negatively charged membranes in response to increases in cellular calcium concentrations in the micromolar concentration range. In addition, human annexin A5 is among the annexins that are localized both extracellularly and intracellularly, as shown in Figure 1. hAnxA5 was discovered in the human placenta in 1979 [1] and is originally believed to be an anticoagulant protein [2] to inhibit intervillous thrombosis and maintain the fluidity of the intervillous circulation.

The most popular name for hAnxA5 is annexin V. In addition to this name, hAnxA5 is also called Anchorin CII [3]; calphobindin-I (CPB I) [2]; endonexin II [4] (E-II); placental anticoagulant protein I (PAP-I) [5]; vascular anticoagulant-alpha (VAC alpha) [6]; lipocortin V [7]; placental protein 4 [8] (PP4) [9]; epididymal secretory protein LI; and thromboplastin inhibitor [10].

hAnxA5 is the most abundant annexin in almost all cells except neurons. This protein is widely distributed in various tissues of the human body [11] and is released into the extracellular space when cells are damaged [12,13]. In cells, AnxA5 is often in a Ca^2+^-dependent manner linked to the regulation of membrane transport, ion channels, Ca^2+^ influx, the cell cycle, apoptosis, and phagocytosis. In addition, substantial amounts of hAnxA5 are found extracellularly, such as in plasma [14], amniotic fluid [15], and cell culture medium [16]. Platelets were the first cells with a physiopathological change in PS asymmetry invoked by the action of agonists such as thrombin or collagen [17]. In vivo, platelets are the classical suppliers of these catalytic sites of the coagulation system. Apoptotic cells also catalyze coagulation. Extracellular hAnxA5 regulates platelets as well as apoptotic cells’ involvement in coagulation and inflammation by modulating the catalytic activity of both as above. Under physiological conditions, it is difficult for procoagulant and inflammatory effects to occur because apoptotic cells are efficiently removed by phagocytes. However, in certain pathological conditions, apoptotic cells occur at a high rate. In these cases, PS, which is heavily exposed on the surface of apoptotic cells, has the potential to promote coagulation and inflammation [18]. Extracellular hAnxA5 may regulate this effect and has selective anticoagulant and anti-inflammatory potential in the interaction of PS-exposed cells with the extracellular environment [19].

The hAnxA5 gene is located at chromosomal region 4*q*26-*q*28 in humans. This gene spans 29 kb, consists of 13 exons and 12 introns, and encodes a single transcript of 1.6 kb [20,21]. hAnxA5 is a single-chain protein with a relative molecular mass of 35,935 Daltons, and it is composed of 320 amino acid residues with an isoelectric point of 4.71 [22]. hAnxA5 contains only one potential glycosylation site, but it is a nonglycosylated protein [23]. When Willems studied its phospholipid binding activity in 1990, it was found that this protein can reversibly bind to membrane structures expressing PS in a calcium ion-dependent manner, and among many phospholipid binding proteins, it has the highest affinity for PS [6]. Studies have shown that hAnxA5 exhibits a high affinity for PS with Kd values ranging from 0.1 to 2 nmol/L [5,6] and rapidly binds to PS in the presence of calcium ions. In addition, it has been reported that hAnxA5 relies on calcium ions for binding to PS, and 1000 µmol/L calcium ion concentration is optimal for its binding activity [24].

Interestingly, hAnxA5 does not have a precursor form, and its protein itself lacks a signal peptide. Therefore, its structural specificity also endows this protein with unique secretory transport mechanisms. A possible explanation for the presence of annexins in circulation might be the release of cytosolic contents from necrotic cells into the surrounding environment. These so-called unconventional secretory pathways, independent of the endoplasmic reticulum and Golgi apparatus, are characterized by insensitivity to inhibitors of ER/Golgi-dependent secretory transport, such as brefeldin A and monensin, and include the release of annexin A1 [25,26,27] and A2 [28,29]. hAnxA5 is released during the apoptosis of cardiomyocytes and THP-1 macrophages [30]. The circumstances under which annexins A4 and A5 are released into the circulation have been less thoroughly investigated [31]. In vitro studies indicated that once released into the circulation, hAnxA5 potentially plays a role in various biochemical and cellular processes involving membranes with PS surface expression.

**Figure 2:**
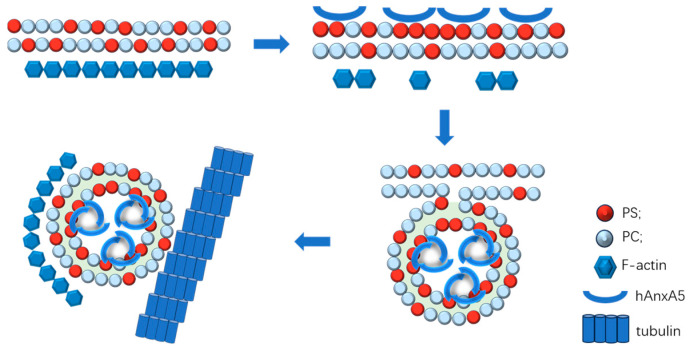
The endocytosis pathway mediated by surface-expressed PS and hAnxA5. Cell surface expression of PS leads to disassembly of the cortical actin network beneath membrane patches exposed to PS. The membrane-facing convex shape of membrane-associated protein A5-trimers and the ability of these trimers to form a two-dimensional lattice drive membrane internalization and bend the membrane patch toward invagination. The invaginated endocytic vesicle membrane structure is closed and wrapped with F-actin, and vesicle membranes are fissioned from the plasma membrane and transported in a microtubule-dependent manner into the cytosol.

When bound to the membrane, hAnxA5 forms trimers of which each monomer retains the convex shape at its phospholipid binding side. The convex shape of phospholipid-bound hAnxA5 trimers induces invagination of part of the membrane using large unilamellar vesicles. hAnxA5 opens a PS-dependent novel portal of cell entry by bending the membrane inward into the cell through two-dimensional (2D) crystallization. hAnxA5 reverses the movement of the PS-expressing membrane patch from blebbing into invagination, which results in vesicle formation and the intracellular trafficking of the endocytic vesicle. The energy required for this action is released by the process of 2D crystallization, as shown in Figure 2. The 2D crystallization of hAnxA5 is the driving force for internalization [32]. The pathway also exists in vivo in cardiomyocytes expressed PS reversibly on the cell surface because of ischemia/reperfusion stress. The novel endocytic pathway of hAnxA5 can be activated during apoptosis and in living tumor cells. The hAnxA5 endocytic pathway does not operate in the same manner in healthy nondisturbed tissues and is an attractive mechanism for targeted delivery and cell entry of drugs designed to kill tumor cells or rescue ischemic/reperfusion-treated cardiomyocytes.

## 2. Tertiary Structure and Polymerization of hAnxA5

**Figure 3:**
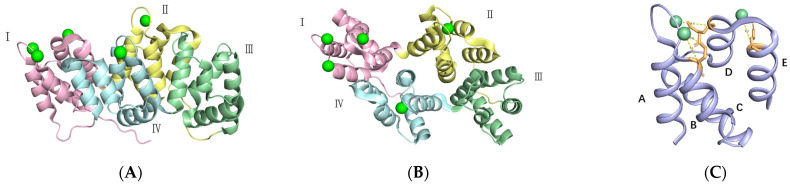
The structural features of hAnxA5. (**A**) Side view (convex face). Calcium atoms are represented as green spheres. Each of the four domains is represented by a different color. (**B**) Top view. (**C**) Close view of domain I showing the five helices, the three bound calcium atoms(green spheres), and three bidentate calcium ligands Glu35 (B site), Glu72 (AB site), and Glu78 (DE site). (Protein Data Bank accession number 1AVR).

hAnxA5 is different from other proteins in the annexin family in terms of sequence and length of amino acid residues, and it has a characteristic amino acid sequence, which is present in the form of a monomer and often exercises biological functions in a polymer. The C-terminal core of hAnxA5 is highly conserved, and its N-terminal amino acid length is about 20 amino acids [33] (Figure 3A). However, the N-terminus plays an important role in the overall structural stability, specific functions, and distribution differences. hAnxA5 is the first determination of crystal structure in the annexin family by Huber and his coworkers [34]. According to the protein structure classification, hAnxA5 is a typical α-type protein with dimensions of 64 × 40 × 30 Å, and the overall structure of the protein is entirely composed of a-helix and random curls, with a typical disc-shaped three-dimensional structure [35]. The disk-like structure consists of a concavo-convex surface; the convex side contains calcium ions and phospholipid junction sites, and the concave side contains the N-terminal head and C-terminal end of the protein. The short N-terminal domain of hAnxA5 determines the structural arrangement of protein regions located on the opposite concave region of the annexin molecule binding Domains I and IV [34,36]. It is suggested that the interaction of the N-terminus with Domain IV on the concave side is transmitted to the loop located in Domain III on the opposite side of the hAnxA5 molecule [37]. The molecule of hAnxA5 is folded into four domains with similar structures, each of which consists of approximately 70 amino acid residues, as shown in Figure 3A,B. Each domain, called the “endonexin folding domain”, consists of five alpha-helices wound into a right-handed superhelix containing a “GXGTXD/E” sequence, yielding a globular structure of approximately 18 Å in diameter [38]. The domains have hydrophobic cores whose amino acid sequences are conserved between the domains and within the annexin family of proteins. The four domains are folded into an almost planar array by tight hydrophobic pair-wise packing of Domains II/III and I/IV to generate modules (II–III) and (I–IV), respectively. The assembly is symmetric with three parallel approximate diads relating II to III, I to IV, and the module (II–III) to (I–IV), respectively [34]. Each one of four homologous tandem annexin repeats forms a typical type II calcium-binding site and five calcium ions are located on the convex face of the molecule of hAnxA5. The AB loop site has the highest affinity for calcium (Figure 3C). Three strongly bound calcium ions are liganded at protruding interhelical loops and Asp or Glu residues in homologous positions in repeats I, II, and IV. The structural features suggest that hAnxA5 attaches to membranes via its convex face through specific calcium-mediated interactions with at least three phospholipids. The Trp185 plays an important role in the Ca^2+^-binding site of repeat III of AnxA5. The domain III induces a large relocation of the Ca^2+^-binding loop regions. Ca^2+^-dependent exposure to the solvent of Trp185, which is buried within the protein core in the absence of calcium, facilitates the interaction between AnxA5 and the phospholipid bilayer. The replacement of Trp185 with alanine decreases the membrane-binding affinity of AnxA5 [39]. Loss of the hydroxyl group (Thr72-->Ala or Thr72-->Lys) causes a reduction in membrane binding, as shown in Table 1.

hAnxA5 molecules, which are monomeric in solution, form 2D crystals at the level of phospholipid surfaces in the presence of Ca^2+^ ions [40]. This property of self-assembly exhibited by hAnxA5 has been suggested to have important biological functional implications. hAnxA5 forms two types of 2D crystals—with either p6 or p3 symmetry—that are both based on annexin trimers [41,42,43]. The domain III is involved in most of the interactions in which hAnxA5 trimers are engaged. Membrane-bound hAnxA5 molecules do not exist as monomers but as trimers and ordered arrays of trimers. There is a viewpoint that the function of hAnxA5 is carried out by trimers or its 2D arrays [44]. The p6 form is observed at low-to-medium (5 to 20%) content of phosphatidylserine (PS) in planar membranes, while the p3 form is observed at high PS content (above 40%). The p6 form forms first and reversible transitions can be induced between the two stable 2D crystalline phases [45]. The basic p6 crystal is the most open and represents the most efficient way to cover a surface with hAnxA5 trimers. When hAnxA5 forms extensive 2D arrays at the level of the cell membrane, the formation of such arrays, controlled by the local activation of Ca^2+^ channels, is likely to affect some membrane properties, such as the diffusion of lipids or/and protein molecules or the mechanical stability of membranes. A variable lattice geometry would allow the hAnxA5 to adapt to local environmental conditions, such as the presence of transmembrane proteins or cytoplasmic filaments, which could prevent the establishment of a unique crystalline arrangement. Another possible advantage provided by this multiplicity of crystal forms could be the ability to support mechanical deformations, which can occur, for example, during cell shape changes [44].

**Table 1:** The functions of certain amino acid residues in *h*AnxA5.

Amino Acid Residues	Position	Function
Trp	Trp-187 in domain Ⅲ	High concentrations of Ca^2+^ bind to Domain Ⅲ of the conserved region and covalent structural changes Trp-187 are exposed [46,47]
Arg	Conserved arginine residues in the endonexin fold of each homology segment	Stabilizing the tertiary structure of annexin A5 [48]
Ser, Thr, special Trp	Thr72,Ser144,Ser228,Ser303,Trp185	Contribute to membrane binding and participate directly in intermolecular contacts with phospholipid membrane components [39]
Asp	Asp-226	A molecular switch of the pH- and Ca^2+^-mediated conformational [49]
Glu	Glu-95	Crucial component of the ion-selectivity filter [50]

## 3. Relevance to and Intervention in Human Physiopathological Disorders

### 3.1. Coagulation and Vascular Abnormalities

Hemostasis is an important physiological response to vascular injury and is activated when blood is exposed to the subendothelial structures or connective tissue. Blood coagulation, as one of the major hemostatic responses, including intrinsic and extrinsic processes, is a cascade of reactions in which inactive precursor proteins are converted into active enzymes. Coagulation factors require the assistance of the corresponding phospholipids to form the appropriate coagulation complexes and promote the coagulation cascade reactions. Among them, PS is the most important phospholipid cofactor [51]. hAnxA5 is abundantly present as an intracellular protein in endothelial cells and platelets and is released in response to tissue injury [52]. Its concentration in the plasma of healthy volunteers is less than 5 ng/mL [14]. Once hAnxA5 in the circulation system binds to PS on the membrane surface, it can self-assemble on the surface of cellular phospholipids to form two-dimensional crystals [53,54], which can effectively segregate PS from other related procoagulant factors, impede phospholipid availability during the formation of key prothrombin complexes, and inhibit the conversion of fibrinogen to fibrin [55,56,57]. hAnxA5 also efficiently inhibits endothelial cell-mediated thrombin formation [58]. These properties are important for the development of drugs for coagulation abnormalities in the clinic.

Moreover, hAnxA5 is also reported to be closely related to cardiovascular disease. Systemic injection of hAnxA5 into mice with damaged blood vessels can effectively reduce vascular inflammation and vascular remodeling and improve vascular function, suggesting that hAnxA5 may play a potential role in the treatment of atherosclerosis (AS) [59]. AS results in an inflammatory state, and plaque rupture is the main cause of cardiovascular disease. Lyso-phosphatidylcholine (LPC) is produced during the oxidation and/or enzymatic modification of low-density lipoprotein (LDL) and is associated with AS. Moreover, hAnxA5 has the potential to reduce vascular inflammation and improve endothelial function in AS animal models [59,60]. Further studies have shown that hAnxA5 reduces the proinflammatory effects of oxidized low-density lipoprotein (OxLDL) and LPC and inhibits macrophage binding and uptake of OxLDL, playing important protective roles in the occurrence of AS and plaque rupture [61]. In a mouse model simulating percutaneous coronary intervention (PCI), it was found that hAnxA5 can inhibit the recruitment of white blood cells and macrophages after vascular injury in a dose-dependent manner, reducing inflammation. Furthermore, hAnxA5 can also reduce vascular intimal hyperplasia and effectively prevent vascular remodeling in a dose-dependent manner [62]. Therefore, hAnxA5 may be used to develop a new treatment strategy for cardiovascular disease.

Other studies have shown that sickle cell disease (SCD) is a kind of genetic blood disease caused by abnormal hemoglobin S (HbS). In patients with SCD, plasma levels of hAnxA5 and the amount of PS-exposed blood cell microparticles are significantly increased [52]. HbS aggregates, causing the erythrocyte membrane to lose its symmetry and twist into a sickle shape, leading to the extracellular expression of PS on the erythrocyte membrane. PS expression on the membrane surface of erythrocytes and circulating microparticles (MPs) plays an important role in the etiology of the hypercoagulable state of SCD, as well as in the reduced red blood cell lifespan and adhesive interactions between erythrocytes and the endothelium. The abnormal adhesion between sickle red cells and activated endothelial cells is partially regulated by PS (erythrocytes) and thrombospondin (endothelial cells). Due to the high-affinity binding between hAnxA5 and PS, which may prevent the interaction between PS and other plasma proteins, hAnxA5 can inhibit the adhesion of sickle red blood cells to the endothelium, regulate exposed PS-induced pathological processes, and lead to heterogeneity in this single gene disease.

### 3.2. Autoimmune Diseases

Antiphospholipid syndrome(APS) is an autoimmune disorder characterized by thrombosis and/or pregnancy loss associated with persistent antiphospholipid antibody(aPL) positivity [63,64,65,66]. hAnxA5 plays a central role in the pathophysiology of APS. The binding of hAnxA5 to the apical membranes of placental villi from APS patients is reduced. The aPL antibodies accelerate coagulation and reduce hAnxA5 anticoagulant activity [67]. The mechanism of the aPL antibodies to accelerate coagulation is probably due to the topographical differences between the binding of the two ligands to phospholipids. Furthermore, hAnxA5 self-assembles on the phospholipid surface, while aPL antibodies disrupt this array structure of hAnxA5, which shields the surface and allows enough space on the phospholipid surface for the binding of coagulation factors such as prothrombin [66]. Current laboratory criteria for APS classification recommend testing positive for aPL. However, a subset of patients with classical APS manifestations test negative. Anti-AnxA5 IgG and IgM levels were significantly correlated with pregnancy morbidity and venous and arterial thrombosis events and showed reasonable sensitivities in their prediction and specificities. Anti-hAnxA5 antibodies are promising for detecting obstetric and thrombotic morbidity in both seropositive- and seronegative-APS patients [68].

### 3.3. Tumorigenesis

hAnxA5 binds to the PS on the surface of tumor vascular endothelial cells, inhibiting tumor angiogenesis and promoting tumor necrosis. In addition, hAnxA5 can also bind to PS on the surface of tumor cells to enhance T-cell-mediated tumor immunity and promote tumor-killing effect [24,69]. hAnxA5 is associated with the progression, metastasis, survival, and prognosis of a variety of cancers.

hAnxA5 can mediate the occurrence of primary hepatocellular carcinoma (HCC) through the integrin and MEK-ERK pathways and has potential applications in the research and treatment of HCC [70]. Compared with those in normal brain tissue, the transcription level of hAnxA5 mRNA and protein expression in glioma increased by 2.45-fold and 2.87-fold, respectively. As the tumor grade increased, the positive rate of hAnxA5 gradually increased, and there was a significant positive correlation between tumor grade and positive rate. Downregulation of hAnxA5 inhibits the growth and motility of glioma cells by inhibiting the Raf/MEK/ERK signaling pathway and induces cell apoptosis [71]. There is also a research report that hAnxA5 promotes GBM cell invasion, MMP-2 expression/activity, and chemoresistance to temozolomide through a PI3K-dependent mechanism [72]. In studies on gastric cancer, it has been found that the hAnxA5 protein can inhibit the proliferation and migration of human gastric cancer MGC-803 cells through exogenous addition, promote cell apoptosis, and, possibly, inhibit cell migration through the regulation of E-cadherin and matrix metalloproteinase-9 (MMP-9). hAnxA5 was able to inhibit the proliferation of HeLa cells by regulating the expression of bcl-2 and bax and inhibit the HeLa cells metastasis by regulating the expression of E-cadherin and MMP-9 [73]. hAnxA5 is highly expressed in breast cancer and is related to the size of the tumor. With the upregulation of hAnxA5, the phosphorylation levels of Raf1, MEK1/2, and ERK1/2 were significantly inhibited, PKC activation was dysregulated, and cell signal transduction was abnormal [74]. These findings suggest that hAnxA5 may become a promising molecular target for clinical gene therapy of breast cancer. The gene and protein levels of hAnxA5 are upregulated in colorectal adenocarcinoma [75], indicating that hAnxA5 is related to tumor staging and the clinical prognosis of colorectal cancer and may be a suitable indicator for predicting tumor progression and adjuvant treatment of colorectal cancer. The expression level of hAnxA5 in lymph node-positive primary bladder tumor tissue was found to be increased, which indicates that hAnxA5 may be related to the development of bladder cancer to the lymph node-positive [76]. HAnxA5 is associated with human skin squamous cell carcinoma. HAnxA5 is upregulated in both skin squamous cell carcinoma cell lines and tissues, indicating that hAnxA5 may be a suitable SCC molecular indicator and drug target for squamous cell carcinoma [77].

hAnxA5 affects the susceptibility to apoptosis and proinflammatory activities of apoptotic cells. There is a viewpoint that the process of tumor progression and metastasis affects the apoptotic potential of a tumor cell population, which directly influences the hAnxA5 expression level in vivo [78].

### 3.4. Pulmonary Fibrosis and Lung Injury

Silicosis is an occupational lung disease caused by long-term inhalation of free silica particles that is characterized by chronic lung inflammation and fibrotic nodules that may lead to progressive pulmonary fibrosis. It is generally believed that activated macrophages are involved in the process of fibrosis through the binding and phagocytosis of silica particles, which stimulates macrophages to produce inflammatory factors and profibrotic cytokines and lead to the overproduction and deposition of extracellular matrix. As an upstream regulator of the Fas/FasL signaling pathway, hAnxA5 is involved in silica-induced activation of macrophages and pulmonary fibrosis via Fas/FasL signaling pathways [79].

Another study on the relevance of hAnxA5 to lung disease reported different experimental results [80]. Acute lung injury (ALI) is a common respiratory syndrome accompanied by an inflammation response. In that study, lipopolysaccharide (LPS) suppressed the viability of alveolar macrophages (AMs) but, interestingly, simultaneously elevated the expression of hAnxA5 in AMs. This study reported that hAnxA5 regulated the expression of TNF-α, interleukin-1β (IL-1β), and interleukin-6 (IL-6). Specifically, the knockdown of hAnxA5 suppressed the expressions of tumor necrosis factor-α (TNF-α), interleukin (IL-1β), and IL-6 and improved the expression levels of inhibitory κB (IκB) and nuclear factor E2-related factor 2 (Nrf2) but inhibited the expression of nuclear transcription factor κB (NF-κB) in comparison with LPS group. This study reported that inhibition of hAnxA5 improved the cell viability and reduced the apoptosis rate compared with the LPS group. This study concluded that loss of hAnxA5 expression attenuates the LPS-induced inflammatory response of rat alveolar macrophages, which suggests that hAnxA5 is a potential anti-inflammatory target in AMs-related inflammation induced by LPS [80].

### 3.5. Liver Disease

Nonalcoholic steatohepatitis (NASH), a common chronic liver disease, is characterized by steatosis, inflammation, and fibrosis. Hepatic macrophages, neutrophils, DC cells, and NK cells are all involved in the development of NASH, with macrophages possibly being more important for steatosis and fibrosis of NASH [81]. hAnxA5 specifically regulates hepatic macrophages by directly interacting with M2-type pyruvate kinase (PKM2) and significantly switches metabolic reprogramming from glycolysis to oxidative phosphorylation in activated macrophages [82]; thus, the steatosis, inflammation, and fibrosis of NASH model mice can be improved, which is conducive to the further treatment of NASH. hAnxA5 targeting PKM2 results in glycolysis inhibition and mitochondrial oxidative metabolism activation, triggering macrophage phenotype shift, and offers a novel therapeutic approach for NASH [83]. These findings provide a new way for the further treatment of NASH.

## 4. Predictability as a Reagent for Detection and Identification

### 4.1. Tumor Biomarkers

hAnxA5 is positively correlated with the development of various tumors and can be used as a marker for disease diagnosis.

In renal cell carcinoma, the PI3K/Akt/mTOR signaling pathway is activated to regulate the epithelial–mesenchymal transition process, and matrix metalloproteinase 2 (MMP2) and MMP9 are also involved. HAnxA5 promotes the proliferation, migration, and invasion of renal cell carcinoma cells [84]. In cervical squamous cell carcinoma, hAnxA5 is associated with the actin gamma 1 (ACTG1) or growth factor receptor-bound protein 2 (Grb2) response, which regulates the apoptosis, proliferation, and differentiation of cancer cells and is considered a potential tumor-specific marker of cervical squamous cell carcinoma [85]. Studies have shown that hAnxA5 is upregulated 134% in primary liver cancer, and hAnxA5 may be a potential marker of portal vein tumor thrombus (PVTT) formation [86]. HAnxA5 has also been proven to be a promising biomarker for colorectal cancer (CRC). Compared with normal tissues, hAnxA5 is upregulated at both the mRNA and protein levels in CRC tissues. Upregulation of hAnxA5 is associated with high tumor staging, liver metastasis, increased recurrence, and decreased overall survival rate. hAnxA5 expression is related to the tumor stage and clinical outcome of CRC. Thus, hAnxA5 could serve as a prognostic marker for tumor progression and assist in the treatment of CRC [75]. In contrast, in a thyroid study, hAnxA5 upregulation was inversely associated with the malignancy of thyroid cancer, suggesting that hAnxA5 might become a useful marker in daily clinical praxis for thyroid cancer patients [87].

In conclusion, hAnxA5 may still be a potential target for diagnostic and treatment strategies targeting tumor stem cells used to explain the development and progression of cancer, as shown in Table 2.

### 4.2. Biomarkers of Neurodegenerative Diseases

Protein misfolding is a central mechanism for the development of neurodegenerative diseases and type 2 diabetes mellitus. HAnxA5 can block the cytotoxic effect of amyloid proteins partly by competitively binding to the membrane surface PS [95] and partly by the direct interaction of hAnxA5 with Aβ, changing the spatial structure of Aβ, which reduces its toxicity caused by its misfolding and amyloid fiber deposition [90].

In AD patients, the level of hAnxA5 in the choroid plexus decreases with the severity of the disease, contrary to Aβ burden and cell death. In the Aβ-treated choroid plexus, hAnxA5 was found to depolarize mitochondria in a Ca^2+^-dependent manner and, thus, play a protective role [91].

In contrast, hAnxA5 levels were significantly increased in the plasma and cerebrospinal fluid of patients with Alzheimer’s disease, and hAnxA5 was associated with the overexpression of mutant human amyloid precursor protein and restored autophagy damage induced by human amyloid protein. These results suggest that hAnxA5 is a potential biomarker for Alzheimer’s disease [96]. It has also been reported that hAnxA5 can interact with Aβ, islet amyloid polypeptide, and α-synuclein inclusions to inhibit toxic amyloid aggregation and protect human islet tissue and islet B cells [90]. Dementia with Lewy bodies (DLB) is clinically and pathologically similar to Parkinson’s disease and Alzheimer’s disease. Lewy bodies of patients are formed due to the abnormal aggregation of α-synuclein, which becomes insoluble. Several studies have reported that the plasma levels of hAnxA5 in DLB patients were also significantly higher than those in normal individuals, indicating that hAnxA5 may be a marker for DLB disease [92]. However, there is no direct evidence that the disturbance of the hAnxA5 protein is related to the disease. Therefore, whether hAnxA5 can be used as a diagnostic or prognostic marker for clinical diseases still needs further studies.

### 4.3. Biomarkers in Heart Failure

Chronic heart failure (CHF) is a syndrome of insufficient cardiac output at rest or after activity caused by various reasons and is often secondary to hypertension, myocardial infarction, atrial fibrillation, cardiomyopathy, and other cardiac diseases. Currently, the world in which we live is going through an increasing number of people becoming at risk of developing hypertensive heart disease. As a result, efficient and rapid identification of patients who are susceptible to developing CHF is going to be of imminent importance. In previous clinical studies, the serum biomarkers, including N-terminal pro-B type natriuretic peptide (NT-proBNP), which is released by stretched myocardial cells, act as a marker in CHF [97].

A study by Ravassa et al. found increased cardiac and plasma levels of hAnxA5 in hypertensive patients with left ventricular hypertrophy or heart failure compared with those in control individuals. They also observed an inverse relationship between the myocardial presence of hAnxA5 and the systolic function of the left ventricle in all hypertensive individuals [98]. In Schurgers’s study, both humans and mice were studied in depth. Using AnxA5 gene knockout mice and wild-type mice as models, it was found that the plasma level of AnxA5 in mice undergoing aortic arch constriction was significantly greater than that in the sham operation group, and hAnxA5 was highly expressed in the lung, kidney, liver, spleen, and other organs, often affected by heart failure. AnxA5 levels were significantly increased in patients with heart failure compared with healthy subjects.

These results show that AnxA5 as a biomarker improves the diagnostic efficiency of conventional biomarkers to predict mortality in HF patients [93].

### 4.4. Biomarkers in Kidney Injury

The immature kidney tissue of premature infants is vulnerable to acute kidney injury(AKI). However, the current biomarkers used for AKI detection are not sensitive or specific, nor are they sufficient to timely detect AKI in preterm infants. A proteomic study of the urine of preterm infants found that 174 of the 1810 proteins identified were selected as the first targeted protein in the discovery phase. A total of 168 proteins were quantified, and the levels of 6 were significantly increased in the AKI group in the verification phase. Through clinical tests, the first urine samples of newborn infants after birth were collected from the biobank for confirmation and verification. The levels of hAnxA5, neutrophil gelatinase-associated lipocalin (NGAL), and protein S100-P were significantly higher in the samples of the first urine samples from patients with AKI than in those from patients without AKI. In summary, levels of the urinary hAnxA5, NGAL, and protein S100-P levels are promising biomarkers for early, accurate prediction of AKI in preterm infants.

Cyclosporin A (CsA) as a calcineurin inhibitor is used effectively to treat children with difficult idiopathic nephrotic syndrome (INS). Prolonged CsA treatment can result in several adverse effects, the most notable of which is nephrotoxicity (CsAN). Uremodulin is considered a marker for distal tubular injury. hAnxA5 is present in the distal tubules. The study showed that the increased urinary excretion of hAnxA5 in children with INS treated with CsA may suggest its usefulness as an early marker in subclinical CsAN [99]. hAnxA5 seems to be a more sensitive indicator of tubular injury in the course of CsA therapy than Uromodulin.

### 4.5. Biomarker in Asthma

Asthma is a common chronic respiratory disease characterized by typical symptoms such as cough, wheezing, and shortness of breath with airflow obstruction and airway remodeling. The hAnxA5 levels were correlated with lung function in patients with asthma and play a potential role in asthma pathogenesis. Using three different allergens or pollutants—ovalbumin (OVA), titanium dioxide (TiO_2_), and house dust mites (Dermatophagoides1, Derp1)—to stimulate model mice, it was found that hAnxA5 expression levels were different in different cells of the trachea and lungs. The study revealed that hAnxA5 levels were lower in patients with asthma than in healthy control subjects, suggesting that asthma decreases the anti-inflammatory effects of hAnxA5. Exacerbation of asthma and pollutant exposure increased hAnxA5 levels, indicating that asthma exacerbation and pollutants can alter the anti-inflammatory action in asthma. The hAnxA5 levels were decreased in the plasma of patients with asthma, and the hAnxA5 levels recovered in patients with exacerbated asthma following pollutant exposure, suggesting that hAnxA5 may be a promising plasma biomarker for the diagnosis of asthma exacerbation and pollutant exposure [100].

## 5. Diagnostic and Therapeutical Usages of hAnxA5

### 5.1. Apoptosis Detector In Vitro or In Vivo

Programmed cell death (PCD) is mandatory for the normal development and tissue homeostasis of adult organisms, which is characterized by a sequence of morphological and biochemical changes; it is of great significance to maintain the normal development and tissue homeostasis of biological organisms [101,102]. Apoptosis, the most abundant form of PCD, involves cell shrinkage, chromatin condensation, membrane blebbing, caspase activation, and the presentation of phosphatidylserine (PS) at the cell surface as an ‘‘eat me’’ signal [103,104]. Methodologies for measuring apoptosis are based on these morphological and biochemical markers; however, most methods can only be applied for in vitro analyses. Biopsies would be required to retrieve information on apoptosis in situ for the analysis of human tissues, which limits the applicability of the current methods in clinical practice.

The ability of hAnxA5 to bind specifically to PS has been identified in vitro and turned into a powerful tool for the detection of apoptosis. hAnxA5 has been used to measure programmed cell death both in vitro and in vivo. hAnxA5 is widely used to identify apoptotic cells and as a probe to detect apoptosis in vivo and in vitro. Assessment of apoptosis would be useful for evaluating the efficacy and mechanisms of therapy and disease progression or regression.

In vitro, compared with other detection methods, FITC-labeled hAnxA5 coupled with PI (FITC-hAnxA5/PI) for the detection of apoptotic cells has easy operation and good repeatability. In molecular imaging, PS has been used as a molecular target for AnxA5 molecular probes in apoptosis imaging techniques.

hAnxA5 has been labeled with ^124^I [105], ^99m^Tc [106,107], ^18^F [108], and ^68^Ga [109] for the development of probes for the imaging and measurement of apoptosis in vivo. In vivo, using FITC-hAnxA5 as a fluorescent probe, apoptosis imaging technology has the characteristics of noninvasive/early/sensitive and specific, which has broad application prospects in clinical practice. The efficacy of novel antitumor compounds that induce apoptosis in solid tumors can be assessed by imaging of radiolabeled hAnxA5 taken up by tumors. ^99m^Tc-hAnxA5 can also be reported to be used for noninvasive apoptosis detection in patients with acute myocardial infarction, intracardiac tumors, and heart transplant rejection. Radionuclide labeling of the hAnxA5 protein is commonly used in clinical practice, and existing research work has systematically reviewed various radiolabeled hAnxA5 probes, including physical half-life, and advantages and disadvantages of probes, such as ease of probe labeling process, cost, background signal, contrast effect, biodistribution in vivo, etc.

Early detection of cellular events is important to predict the outcome of patients. An imaging technique was developed to measure and monitor tumor cells undergoing programmed death caused by radiation and chemotherapy using ^99m^Tc-EC-hAnxA5 [107]. Ethylenedicysteine (EC) was conjugated to hAnxA5 using sulfo-N-hydroxysuccinimide and 1-ethyl-3-(3-dimethylaminopropyl) carbodiimide-HCl as coupling agents. In vitro cellular uptake, pre- and postradiation (10–30 Gy), and paclitaxel treatment were quantified using ^99m^Tc-EC-hAnxA5. Planar images confirmed that the tumors could be visualized clearly with ^99m^Tc-EC-hAnxA5. Apoptosis can be quantified using ^99m^Tc-EC-hAnxA5, and it is feasible to use ^99m^Tc-EC-hAnxA5 to image tumor apoptosis. ^99m^Tc-EC hAnxA5 has been suggested to be a candidate for evaluating baseline and posttreatment-induced apoptosis in cancer patients [110].

### 5.2. hAnxA5-Associated Targeted Drug Delivery (TDD) Strategy

**Figure 4:**
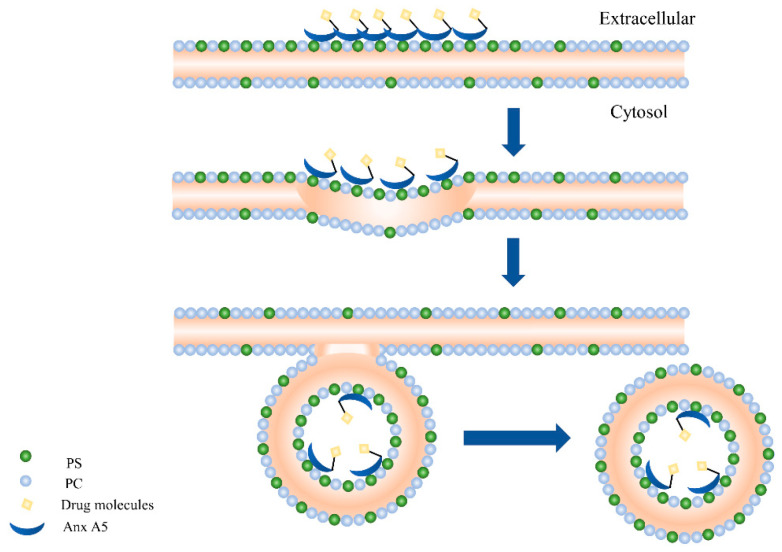
hAnxA5-based phosphatidylserine-dependent endocytosis for the targeted therapeutic agent. hAnxA5 binds to PS, which is everted on the cell membrane. Then, hAnxA5 forms two-dimensional crystals that bend the plasma membrane. Next, endocytic vesicles form. Finally, vesicles in the plasma membrane are divided by an as-yet unidentified mechanism.

The targeted drug delivery (TDD) strategy was used to minimize harmful side effects while effectively treating diseases. The formation of targeted drugs using hAnxA5 as a carrier is mainly due to the eversion of PS in cells, and PS exposed on the cell surface is an effective target of the TDD strategy. For example, endothelial cells in the tumor vascular system express a large amount of PS [111]. By chemically cross-linking or fusing hAnxA5 with enzyme molecules, a “specific molecular missile” for the targeted search of PS was constructed (see Fig TDD) [70]. PS exposure is not only present in apoptotic, primary, and secondary necrotic cells but also observed on the surface of certain living cells. However, these cells do not trigger phagocytosis by phagocytes, so PS can be a target for hAnxA5 drug chimeras. When the two are connected, the internalization of hAnxA5 enables specific drug delivery to cells expressing PS, as shown in Figure 4. Therefore, hAnxA5 can be used as a carrier to construct targeted drugs, and its prospect is also very considerable. For example, some studies have coupled doxorubicin with AnxA5, and its tumor-killing effect is stronger than that of either agent alone, suggesting that AnxA5 may enhance the antitumor effect of doxorubicin by targeting tumor vascular endothelial cells and tumor cells [112].

### 5.3. hAnx5-Associated Anticancer Treatment

Immunological tolerance in the periphery is constantly being maintained by dendritic cells processing material from apoptotic cells (ACs) in a steady state. hAnxA5 translocates to the surface of ACs to function as redundant tolerogenic signals in vitro and in vivo. Exposure of bone marrow-derived dendritic cells to hAnxA5 in vitro results in the inhibition of both proinflammatory cytokine secretion and the upregulation of costimulatory molecules upon TLR stimulation [113]. In vivo, co-injection of OVA-expressing and hAnxA5-expressing ACs into OVA-immunoreactive mice prevented the induction of Ag-specific CD8(+) T cells. Mice immunized with hAnxA5-expressing ACs became refractory to antigenic challenge. These results suggest that hAnxA5 contributes to the AC-induced suppression of dendritic cell activation. It suggests that manipulating hAnxA5-mediated immunosuppression may prove beneficial for patients with cancer, autoimmune diseases, or chronic inflammatory disorders. In addition, Dectin-1, a member of the C-type lectin receptor (CLR) superfamily expressed on dendritic cells, macrophages, and other immune cells [114], is a key tolerance receptor with nanomolar affinity for the core domain of hAnxA5 exposed to apoptotic cells. Thus, it becomes an important immune checkpoint system and provides a link between the immunosuppressive signaling of apoptotic cells and the maintenance of peripheral immune tolerance [115].

In recent years, immune therapy has been used as a popular tool for the treatment of cancer, and its mechanism is mainly aimed at the body’s immune status. Low or hyperfunction artificially enhances or inhibits the body’s immune response to achieve the goal of treating a disease. Tumor immunotherapy aims to activate the body’s immune system and relies on its immune function to kill cancer cells and tumor tissue. Cancer immunotherapy, especially therapeutic vaccination, usually does not produce a strong antitumor immune response. Recently, it has been found that the intratumoral burst release of the protein hAnxA5 from intravenously injected hollow mesoporous nanoparticles made of diselenide-bridged organosilica generates robust antitumor immunity by exploiting the the capacity of primary tumors to act as antigen depots [116]. hAnxA5 blocks immunosuppressive apoptosis and promotes immunostimulatory secondary necrosis by binding to the phagocytic marker phosphatidylserine on dying tumor cells. In mice bearing large established tumors, the burst release of hAnxA5 induced systemic cytotoxic T-cell responses and immunological memory associated with tumor regression and the prevention of relapse. Reducing apoptosis signaling via in situ vaccination could be a novel and versatile strategy for the generation of adaptive antitumor immune responses.

In the tumor microenvironment, PS exposure is significantly increased on the surface of tumor cells or tumor cell-derived microvesicles, which have innate immunosuppressive properties that promote tumor growth and metastasis [117]. hAnxA5 disturbs the PS-dependent clearance of dying tumor cells by macrophages and leads to the accumulation of the secondary necrotic cells and increased uptake of the dead cells by dendritic cells. hAnxA5 should be used as an immune stimulator and can be combined with irradiation, chemotherapy, and hyperthermia to induce immunogenic cell death forms in vivo or ex vivo [118]. The interactions between immune cells and PS molecules exposed on the surface of apoptotic tumor bodies, such as chemotherapy-induced interactions, contribute to the formation of an immunosuppressive tumor microenvironment (TME). hAnxA5 binds with high affinity to PS externalized by apoptotic cells, thus hindering the interaction of these cells with immune cells. hAnxA5 preferentially homed to the TME enriched with PS-exposed tumor cells, and the fusion of tumor antigen peptide with hAnxA5 significantly enhances its immunogenicity and antitumor efficacy in vivo when administered after chemotherapy [119]. hAnxA5 administration rescues the immunosuppressive state of the TME induced by chemotherapy. In addition, the antitumor therapeutic effects of the hAnxA5-antigen peptide can be further enhanced by the administration of other immune checkpoint inhibitors. These findings support the administration of hAnxA5 as a promising immune checkpoint inhibitor for cancer treatment after chemotherapy.

### 5.4. hAnx5-Associated Thrombotic Diseases Treatment

hAnxA5 is widely used in anticoagulation and antithrombotic therapy. PS expressed by platelets supports the assembly of the prothrombin enzyme complex, which consists of factor Va (FVa), factor Xa (FXa), and prothrombin. hAnxA5 competes with FVa, FXa, and prothrombin for PS binding, thus preventing the formation of the prothrombin enzyme complex. In an animal model of arterial embolism, the concentration of radionuclide-labeled hAnxA5 in thrombi was reportedly 13 times greater than that in controls [120]. When platelets are activated by thrombin or collagen, the number of binding sites between hAnxA5 and platelets increases 15–20 times [14].

Two hAnxA5-based thrombolytic agents have been reported, both of which are capable of targeting platelet-containing thrombi [121]. These two thrombolytics showed the same specific activity as native hAnxA5 when bound to the PS cell membrane. The important parameters in vitro, such as activated fibrinolytic kinetic parameters and in vitro thrombolytic activity were almost identical to those of wild-type thrombolytic agents. This study demonstrated the feasibility of using hAnxA5 as a thrombolytic component to construct a chimeric thrombolytic agent. The conjugate of hAnxA5 and the urokinase B chain at the stoichiometric ratio of 1:1 has the same catalytic activity as urokinase and similar plasminogen activation activity, and the affinity of the conjugate for the PS membrane is the same as that of hAnxA5 [122]. However, in the rat model of pulmonary embolism, this conjugate showed 3 to 4 times greater in vivo thrombolytic activity than urokinase, and the plasma clearance rate was basically the same as that of urokinase or its B chain. A chimeric protein composed of the extracellular region of tissue factor (TF) or soluble sTF and hAnxA5 showed biphasic effects during blood coagulation: at low concentrations, it accelerated blood clotting; at high concentrations, it acted as an anticoagulant [123]. The sTF-hAnxA5 chimera is a targeted procoagulase protein that can be used to accelerate thrombin production in the case of PS exposure in the vascular system, such that may occur at the site of injured vascular sites or within the tumor vasculature. A hAnxA5 homodimer, DAV (73 kDa), which has a half-life of 6.5 h in the rat circulation, was constructed [124]. DAV has a higher cellular affinity for exposed PS than hAnxA5. DAV has been found to effectively inhibit prothrombin complex activity and to reduce the formation of mediators of coagulation and reperfusion injury. Based on hAnxA5 and the snake venom-derived antiplatelet peptide ECH, a novel multi-mechanism-coupled protein was designed and constructed [125]. The fusion protein exhibited multi-targeted binding characteristics, combined the dual properties of integrin regulation and PS binding, and showed good antithrombotic activity in vivo. Nanocarriers containing the thrombolytic drug lumbrokinase were chemically conjugated to hAnxA5 [126]. It was found that lumbrokinase-loaded targeted micelles had stronger thrombolysis potency than free lumbrokinase and LK-loaded nontargeted micelles. The hAnxA5 conjugate micelles could be a potential targeted thrombolytic drug delivery system. Interestingly, a thrombin-derived prothrombotic conjugate targeting phosphatidylserine based on hAnxA5 was also established to develop a vascular targeting treatment for brain arteriovenous malformations [127]. The hAnxA5-thrombin conjugate induced rapid thrombosis (fibrin deposition) on irradiated endothelial cells under shear stress in the parallel-plate flow device. Unconjugated, non-targeting thrombin did not induce fibrin deposition.

### 5.5. Application in Systemic Lupus Erythematosus (SLE) Treatment

SLE is an autoimmune disease. Patients with SLE have an autoantibody, aPL, which can react with cardiolipin, PS, and their protein ligands, such as glycoprotein I and hAnxA5. Antiphospholipid antibodies in SLE patients competitively bind to PS with hAnxA5, which significantly reduces the level of hAnxA5 that can bind to the membrane structure with exposed PS, thus significantly reducing the antithrombotic and anti-inflammatory effects of hAnxA5. Clinically, SLE patients are much more likely to suffer from inflammatory diseases and cardiovascular diseases than the normal population. However, this may point to a way to effectively treat or reduce the damage of SLE/aPL patients by interfering with hAnxA5 levels in vivo [128]. Hydroxychloroquine, an antimalarial drug, is used in the treatment of SLE. Hydroxychloroquine inhibits GPIIb/IIIa expression on aPL-activated platelets, prevents the aPL-induced disruption of hAnxA5, and reverses the formation of aPL-β_2_GPI-PL bilayer complexes [129]. Hydroxychloroquine restores hAnxA5 expression and reduces IgG binding to syncytiotrophoblasts, reversing the effects of the aPL [130].

### 5.6. Treatment of EIB in Asthma

Asthma is a chronic disease characterized by airway inflammation, intermittent symptoms, and airflow obstruction, and mediators such as histamine, leukotrienes, and prostanoids, released from the mast cells and epithelial cells in response to a hyperosmolar stimulus, may be responsible for the bronchospasm [131]. One research on exercise-induced bronchoconstriction (EIB) in asthma has been conducted to evaluate exhaled breath condensate (EBC) hAnxA5 levels in EIB of asthmatic children. There was an inverse correlation between hAnxA5 levels and a reduction in forced expiratory volume at one second percent (FEV 1%) (*p* = 0.009, *r* = −0.598). This study showed that the EBC hAnxA5 may have a possible preventive role in EIB in asthma. hAnxA5 and related compounds may provide novel therapeutic approaches to the treatment of EIB in asthma [132].

## 6. Conclusions and Perspectives

In this article, we systematically review the multiple roles of the hAnxA5 in physiopathology. The understanding of the characteristics and mechanism of hAnxA5 in physiological and pathological phenomena of the human body has been greatly expanded with the development of a large number of studies. As a unique and important member of the multigene annexin family of phospholipid-binding proteins, hAnxA5 has a unique self-assembly mode, which distinguishes it from many other proteins of the same molecular weight size. Phospholipids play a special role in the body. As a protein that specifically binds to phosphatidylserine, an important signaling molecule, hAnxA5, exhibits diverse biological functions and participates in numerous physiological and pathological processes.

Although hAnxA5 is not a giant protein molecule and has a molecular weight of about 36 kDalton, so far, there are no reports about the truncated form or small molecule mimics of hAnxA5 with full biological activity. This may be related to the special structural properties of hAnxA5. Thus, further research on the modification of the structure of hAnxA5 as a therapeutical candidate in clinical practice may still need to be developed. The dimer prolongs the half-life of AnxA5 in vivo, which is positive information that contributes to medical applications.

Second, with the expanded understanding of the antithrombotic mechanism of hAnxA5, it should be qualified as a factor of the endogenous anticoagulation system in vivo. In addition to the particularly important mechanical shielding effect based on phosphatidylserine, hAnxA5 may also be able to downregulate the expression of tissue factors or interact with other ligands involved in hemostasis, such as sulfa peptide and heparin, or upregulate urokinase-type plasminogen activator or exhibit weak interaction with other lipid molecules, such as sulfatide and others. hAnxA5 may have more complex and refined coagulation regulatory functions.

Some patients with arterial thrombosis have antibodies against hAnxA5 in vivo. However, the detailed mechanism of action remains unclear. Further research is needed to elucidate the precise mechanism of action of hAnxA5 and its antibodies in arterial thrombosis. The cross-reaction effect of hAnxA5 is also worthy of investigation in the future.

Recently, there have been reports that hAnxA5 may be a promising agent for the treatment of severe COVID-19 patients. The severe forms of COVID-19 include endothelial damage, blood vessel leakage, blood pressure decrease, clot formation, microthrombi, and multiple organ failure, which indicates a central role of the vascular endothelium. Submicron extracellular vesicles, called microparticles (MPs), are described in several diseases as being involved in proinflammatory pathways [133,134]. MPs from intubated COVID-19 patients not only significantly increased the proportion of endothelial cell death but also induced endothelial expression of proadhesive proteins. MPs from intubated COVID-19 patients induced P-selectin expression by endothelial cells. hAnxA5 prevents the binding of MPs with ECs [133] and abolishes MP-induced EC death, VCAM-1 or P-selectin overexpression, abnormal neutrophil adhesion, and netosis [135].

To some extent, hAnxA5 has been widely used in diagnosis and medical applications. hAnxA5 can be regarded as a molecule that plays a core role. Although hAnxA5 participates in many physiopathological regulations, many of them remain unclear or investigated insufficiently, and further studies need to be carried out not only to promote a deep understanding of its functional properties and mechanism but also to expand its biomedical application.

Additional important physiological functions may be discovered in the future, as this family of proteins has been highly conserved during evolution and most organisms contain multiple members of the family that may act as potential backup proteins. However, there is still a long way to go to understand the precise functions of individual annexins and it may prove difficult to take into account the potential redundancy of annexin functions, their involvement in multicomponent membrane-associated scaffolds, and their interaction with several signaling pathways.

## Figures and Tables

**Table 2:** Predictability of *h*AnxA5 as a biomarker for certain human diseases.

Biomarker Type	Diseases	Content Change	*h*AnxA5 Mechanism of Action	References
Cancer biomarker	RCC	↑	Activates PI3K/Akt/mTOR pathway; promotes the expression of MMP2 and MMP9	[84]
	Gastric carcinoma	↑	Activation of MRP promotes resistance to gastric cancer.	[88]
	NPC	↑	The mechanism remains uncertain.	[89]
	Thyroid carcinoma	↓	The mechanism remains uncertain.	[87]
	PVTT	↑	The mechanism remains uncertain.	[86]
	CRC	↑	The mechanism remains uncertain.	[75]
Neurodegenerative diseases biomarker	PD	Choroid plexus↓ Cerebrospinal fluid↑	The mechanism remains uncertain.	[90]
	AD	↑	Ca^2+^ restores mitochondrial depolarization.	[91]
	DLB	↑	Modulates alpha-synuclein.	[92]
Other diseases biomarker	Bronchial asthma	↑	Modulates TGF-β1, connective tissue growth factor.	
	Heart failure	↑	The systolic function of the left ventricle was negatively correlated.	[93]
	Acute kidney injury in preterm infants	↑	Urinary *h*AnxA5 is higher in the early stages.	[94]

RCC, renal cell carcinoma; NPC, nasopharyngeal carcinoma; PVTT, portal vein tumor thrombus; CRC, colorectal cancer; AD, Alzheimer’s disease; PD, Parkinson’s disease; DLB, dementia with Lewy bodies; MRP, multidrug resistance protein.

## Data Availability

All data are available from the author upon reasonable request.

## References

[1] Bohn H., Kraus W. (1979). Isolierung and charakterisierung eines neuen plazentaspezifischen proteins (PP10). Arch. Gynecol..

[2] Kume K. (1989). Isolation of calphobindin-II and its mechanism of anticoagulant activity. Nihon Sanka Fujinka Gakkai Zasshi.

[3] Mollenhauer J., Bee J.A., Lizarbe M.A., von der Mark K. (1984). Role of anchorin CII, a 31,000-mol-wt membrane protein, in the interaction of chondrocytes with type II collagen. J. Cell Biol..

[4] Schlaepfer D.D., Mehlman T., Burgess W.H., Haigler H.T. (1987). Structural and functional-characterization of endonexin ii, a calcium-binding and phospholipid-binding protein. Proc. Natl. Acad. Sci. USA.

[5] Tait J.F., Gibson D., Fujikawa K. (1989). Phospholipid binding properties of human placental anticoagulant protein-I, a member of the lipocortin family. J. Biol. Chem..

[6] Andree H.A., Reutelingsperger C.P., Hauptmann R., Hemker H.C., Hermens W.T., Willems G.M. (1990). Binding of vascular anticoagulant alpha (VAC alpha) to planar phospholipid bilayers. J. Biol. Chem..

[7] Pepinsky R.B., Tizard R., Mattaliano R.J., Sinclair L.K., Miller G.T., Browning J.L., Chow E.P., Burne C., Huang K.S., Pratt D. (1988). Five distinct calcium and phospholipid binding proteins share homology with lipocortin I. J. Biol. Chem..

[8] Inaba N., Shirotake S., Ota Y., Fukazawa I., Nito A., Ijichi M., Takamizawa H., Bohn H. (1986). Clinical significance of a new membrane associated placental protein 4 (PP4) in gynecologic malignancies. Nihon Sanka Fujinka Gakkai Zasshi.

[9] Grundmann U., Abel K.J., Bohn H., Löbermann H., Lottspeich F., Küpper H. (1988). Characterization of cDNA encoding human placental anticoagulant protein (PP4): Homology with the lipocortin family. Proc. Natl. Acad. Sci. USA.

[10] Shidara Y., Takahashi O., Murata M., Kume K., Narita M., Sato H., Sato Y., Higuchi M., Maki M. (1989). Molecular structure and function of tissue thromboplastin and thromboplastin inhibitor, calphobindin in blood coagulation and fibrinolysis. Nihon Rinsho.

[11] Heerde W., Lap P., Schoormans S., Groot P.G., Reutelingsperger C., Vroom T.M. (2004). Localization of annexin A5 in human tissues. Annexins.

[12] Kaneko N., Matsuda R., Hosoda S., Kajita T., Ohta Y. (1996). Measurement of plasma annexin V by ELISA in the early detection of acute myocardial infarction. Clin. Chim. Acta.

[13] Peetz D., Hafner G., Blankenberg S., Peivandi A.A., Schweigert R., Brunner K., Dahm M., Rupprecht H.J., Möckel M. (2002). Annexin V does not represent a diagnostic alternative to myoglobin for early detection of myocardial infarction. Clin. Lab..

[14] Flaherty M.J., West S., Heimark R.L., Fujikawa K., Tait J.F. (1990). Placental anticoagulant protein-I: Measurement in extracellular fluids and cells of the hemostatic system. J. Lab. Clin. Med..

[15] Römisch J., Schuler E., Bastian B., Bürger T., Dunkel F.G., Schwinn A., Hartmann A.A., Pâques E.P. (1992). Annexins I to VI: Quantitative determination in different human cell types and in plasma after myocardial infarction. Blood Coag. Fibrinol..

[16] Koster J.J., Boustead C.M., Middleton C.A., Walker J.H. (1993). The sub-cellular localization of annexin V in cultured chick-embryo fibroblasts. Biochem. J..

[17] Bevers E.M., Comfurius P., van Rijn J.L., Hemker H.C., Zwaal R.F. (1982). Generation of prothrombin converting activity and the exposure of phosphatidylserine at the outer surface of platelets. Eur. J. Biochem..

[18] Holmgren L., O’Reilly M.S., Folkman J. (1995). Dormancy of micrometastasis: Balanced proliferation and apoptosis in the presence of angiogenesis suppression. Nat. Med..

[19] Bennett M.R., Gibson D.F., Schwartz S.M., Tait J.F. (1995). Binding and phagocytosis of apoptotic vascular smooth muscle cells is mediated in part by exposure of phosphatidylserine. Circ. Res..

[20] Cookson B.T., Engelhardt S., Smith C., Bamford H.A., Prochazka M., Tait J.F. (1994). Organization of the human annexin V (ANX5) gene. Genomics.

[21] Fernández M.P., Morgan R.O., Fernández M.R., Carcedo M.T. (1994). The gene encoding human annexin V has a TATA-less promoter with a high G+C content. Gene.

[22] Funakoshi T., Heimark R.L., Hendrickson L.E., Mcmullen B.A., Fujikawa K. (1987). Human placental anticoagulant protein: Isolation and characterization. Biochemistry.

[23] Raynal P., Pollard H.B. (1994). Annexins: The problem of assessing the biological role for a gene family of multifunctional calcium- and phospholipid-binding proteins. Biochim. Biophys. Acta.

[24] van Genderen H.O., Kenis H., Hofstra L., Narula J., Reutelingsperger C.P. (2008). Extracellular annexin A5: Functions of phosphatidylserine-binding and two-dimensional crystallization. Biochim. Biophys. Acta.

[25] Philip J.G., Flower R.J., Buckingham J.C. (1998). Blockade of the classical pathway of protein secretion does not affect the cellular exportation of lipocortin 1. Regul. Pept..

[26] Solito E., Mulla A., Morris J.F., Christian H.C., Flower R.J., Buckingham J.C. (2003). Dexamethasone induces rapid serine-phosphorylation and membrane translocation of annexin 1 in a human folliculostellate cell line via a novel nongenomic mechanism involving the glucocorticoid receptor, protein kinase C, phosphatidylinositol 3-kinase, and mitogen-activated protein kinase. Endocrinology.

[27] Omer S., Meredith D., Morris J.F., Christian H.C. (2006). Evidence for the role of adenosine 5’-triphosphate-binding cassette (ABC)-A1 in the externalization of annexin 1 from pituitary folliculostellate cells and ABCA1-transfected cell models. Endocrinology.

[28] Peterson E.A., Sutherland M.R., Nesheim M.E., Pryzdial E.L. (2003). Thrombin induces endothelial cell-surface exposure of the plasminogen receptor annexin 2. J. Cell Sci..

[29] Deora A.B., Kreitzer G., Jacovina A.T., Hajjar K.A. (2004). An annexin 2 phosphorylation switch mediates p11-dependent translocation of annexin 2 to the cell surface. J. Biol. Chem..

[30] Ravassa S., Bennaghmouch A., Kenis H., Lindhout T., Hackeng T., Narula J., Hofstra L., Reutelingsperger C. (2005). Annexin A5 down-regulates surface expression of tissue factor: A novel mechanism of regulating the membrane receptor repertoir. J. Biol. Chem..

[31] Masuda J., Takayama E., Satoh A., Ida M., Shinohara T., Kojima-Aikawa K., Ohsuzu F., Nakanishi K., Kuroda K., Murakami M. (2004). Levels of annexin IV and V in the plasma of pregnant and postpartum women. Thromb. Haemost..

[32] Kenis H., van Genderen H., Bennaghmouch A., Rinia H.A., Frederik P., Narula J., Hofstra L., Reutelingsperger C.P. (2004). Cell surface-expressed phosphatidylserine and annexin A5 open a novel portal of cell entry. J. Biol. Chem..

[33] Burgmaier M., Schutters K., Willems B., van der Vorst E.P., Kusters D., Chatrou M., Norling L., Biessen E.A., Cleutjens J., Perretti M. (2014). AnxA5 reduces plaque inflammation of advanced atherosclerotic lesions in apoE(-/-) mice. J. Cell Mol. Med..

[34] Huber R., Römisch J., Paques E.P. (1990). The crystal and molecular structure of human annexin V, an anticoagulant protein that binds to calcium and membranes. EMBO J..

[35] Benz J., Hofmann A. (1997). Annexins: From structure to function. Biol. Chem..

[36] Swairjo M.A., Concha N.O., Kaetzel M.A., Dedman J.R., Seaton B.A. (1995). Ca(2+)-bridging mechanism and phospholipid head group recognition in the membrane-binding protein annexin V. Nat. Struct. Biol..

[37] Lizarbe M.A., Barrasa J.I., Olmo N., Gavilanes F., Turnay J. (2013). Annexin-phospholipid interactions. Functional implications. Int. J. Mol. Sci..

[38] Liemann S., Huber R. (1997). Three-dimensional structure of annexins. Cell Mol. Life Sci..

[39] Campos B., Mo Y.D., Mealy T.R., Li C.W., Swairjo M.A., Balch C., Head J.F., Retzinger G., Dedman J.R., Seaton B.A. (1998). Mutational and crystallographic analyses of interfacial residues in annexin V suggest direct interactions with phospholipid membrane components. Biochemistry.

[40] Concha N.O., Head J.F., Kaetzel M.A., Dedman J.R., Seaton B.A. (1992). Annexin V forms calcium-dependent trimeric units on phospholipid vesicles. FEBS Lett..

[41] Voges D.D., Berendes R., Burger A., Demange P., Baumeister W., Huber R. (1994). Three-dimensional structure of membrane-bound annexin V. A correlative electron microscopy-X-ray crystallography study. J. Mol. Biol..

[42] Reviakine I., Bergsma-Schutter W., Mazères-Dubut C., Govorukhina N., Brisson A. (2000). Surface topography of the p3 and p6 annexin V crystal forms determined by atomic force microscopy. J. Struct. Biol..

[43] Hong S., Na S., Kim O.H., Jeong S., Oh B.C., Ha N.C. (2020). High-resolution structures of annexin A5 in a two-dimensional array. J. Struct. Biol..

[44] Oling F., Bergsma-Schutter W., Brisson A. (2001). Trimers, dimers of trimers, and trimers of trimers are common building blocks of annexin a5 two-dimensional crystals. J. Struct. Biol..

[45] Reviakine I., Bergsma-Schutter W., Morozov A., Brisson A. (2001). Two-dimensional crystallization of annexin A5 on phospholipid bilayers and monolayers: A solid-solid phase transition between crystal forms. Langmuir.

[46] Concha N.O., Head J.F., Kaetzel M.A., Dedman J.R., Seaton B.A. (1993). Rat Annexin V Crystal structure: Ca^2+^-induced conformational changes. Science.

[47] Lewit-Bentley A., Morera S., Huber R., Bodo G. (1992). The effect of metal binding on the structure of annexin V and implications for membrane binding. Eur. J. Biochem..

[48] Gerke V., Moss S.E. (2002). Annexins: From Structure to Function. Physiol. Rev..

[49] Sopkova-De Oliveira Santos J., Vincent M., Tabaries S., Chevalier A., Kerbœuf D., Russo-Marie F., Lewit-Bentley A., Gallay J. (2001). Annexin A5 D226K structure and dynamics: Identification of a molecular switch for the large-scale conformational change of domain III. FEBS Lett..

[50] Berendes R., Voges D., Demange P., Huber R., Burger A. (1993). Structure-Function Analysis of the Ion Channel Selectivity Filter in Human Annexin V. Science.

[51] Lentz B.R. (2003). Exposure of platelet membrane phosphatidylserine regulates blood coagulation. Prog. Lipid Res..

[52] van Tits L.J., van Heerde W.L., Landburg P.P., Boderie M.J., Muskiet F.A., Jacobs N., Duits A.J., Schnog J.B. (2009). Plasma annexin A5 and microparticle phosphatidylserine levels are elevated in sickle cell disease and increase further during painful crisis. Biochem. Biophys. Res. Commun..

[53] Mosser G., Ravanat C., Freyssinet J.M., Brisson A. (1991). Sub-domain structure of lipid-bound annexin-V resolved by electron image analysis. J. Mol. Biol..

[54] Andree H.A., Stuart M.C., Hermens W.T., Reutelingsperger C.P., Hemker H.C., Frederik P.M., Willems G.M. (1992). Clustering of lipid-bound annexin V may explain its anticoagulant effect. J. Biol. Chem..

[55] Rand J.H., Wu X.X., Quinn A.S., Taatjes D.J. (2008). Resistance to annexin A5 anticoagulant activity: A thrombogenic mechanism for the antiphospholipid syndrome. Lupus.

[56] Wahezi D.M., Ilowite N.T., Wu X.X., Pelkmans L., Laat B., Schanberg L.E., Rand J.H. (2013). Annexin A5 anticoagulant activity in children with systemic lupus erythematosus and the association with antibodies to domain I of β2-glycoprotein I. Lupus.

[57] Arnold P., Lu X., Amirahmadi F., Brandl K., Arnold J.M., Feng Q. (2014). Recombinant human annexin A5 inhibits proinflammatory response and improves cardiac function and survival in mice with endotoxemia. Crit. Care Med..

[58] van Heerde W.L., Poort S., van ‘t Veer C., Reutelingsperger C.P., de Groot P.G. (1994). Binding of recombinant annexin V to endothelial cells: Effect of annexin V binding on endothelial-cell-mediated thrombin formation. Biochem. J..

[59] Ewing M.M., de Vries M.R., Nordzell M., Pettersson K., de Boer H.C., van Zonneveld A.J., Frostegård J., Jukema J.W., Quax P.H. (2011). Annexin A5 therapy attenuates vascular inflammation and remodeling and improves endothelial function in mice. Arterioscler. Thromb. Vasc. Biol..

[60] Cederholm A., Frostegård J. (2005). Annexin A5 in cardiovascular disease and systemic lupus erythematosus. Immunobiology.

[61] Domeij H., Hua X., Su J., Bäcklund A., Yan Z., Frostegård A.G., Haeggström J.Z., Modéer T., Frostegård J. (2013). Annexin A5 inhibits atherogenic and pro-inflammatory effects of lysophosphatidylcholine. Prostaglandins Other Lipid Mediat..

[62] Ewing M.M., Karper J.C., Sampietro M.L., de Vries M.R., Pettersson K., Jukema J.W., Quax P.H. (2012). Annexin A5 prevents post-interventional accelerated atherosclerosis development in a dose-dependent fashion in mice. Atherosclerosis.

[63] Alijotas-Reig J., Esteve-Valverde E., Anunciación-Llunell A., Marques-Soares J., Pardos-Gea J., Miró-Mur F. (2022). Pathogenesis, Diagnosis and Management of Obstetric Antiphospholipid Syndrome: A Comprehensive Review. J. Clin. Med..

[64] Rand J.H., Wu X.X., Quinn A.S., Taatjes D.J. (2010). The annexin A5-mediated pathogenic mechanism in the antiphospholipid syndrome: Role in pregnancy losses and thrombosis. Lupus.

[65] Khamashta M., Taraborelli M., Sciascia S., Tincani A. (2016). Antiphospholipid syndrome. Best Pract. Res. Clin. Rheumatol..

[66] Singh N.K., Yadav D.P., Gupta A., Singh U., Godara M. (2013). Role of anti-annexin A5 in pathogenesis of hypercoagulable state in patients with antiphospholipid syndrome. Int. J. Rheum. Dis..

[67] Vogt E., Ng A.K., Rote N.S. (1997). Antiphosphatidylserine antibody removes annexin-V and facilitates the binding of prothrombin at the surface of a choriocarcinoma model of trophoblast differentiation. Am. J. Obstet. Gynecol..

[68] Omar G., Mohamed F.I., Sadek H.A., Mamdouh A.S. (2017). Diagnostic value of anti-annexin A5 antibodies in seropositive versus seronegative antiphospholipid syndrome patients. Egypt. Rheumatol..

[69] Stach C.M., Turnay X., Voll R.E., Kern P.M., Kolowos W., Beyer T.D., Kalden J.R., Herrmann M. (2000). Treatment with annexin V increases immunogenicity of apoptotic human T-cells in Balb/c mice. Cell Death Differ..

[70] Sun X., Liu S., Wang J., Wei B., Guo C., Chen C., Sun M.Z. (2018). Annexin A5 regulates hepatocarcinoma malignancy via CRKI/II-DOCK180-RAC1 integrin and MEK-ERK pathways. Cell Death Dis..

[71] Truong D., Fiorelli R., Barrientos E.S., Melendez E.L., Sanai N., Mehta S., Nikkhah M. (2019). A three-dimensional (3D) organotypic microfluidic model for glioma stem cells—Vascular interactions. Biomaterials.

[72] Wu L., Yang L., Xiong Y., Guo H., Shen X., Cheng Z., Zhang Y., Gao Z., Zhu X. (2014). Annexin A5 promotes invasion and chemoresistance to temozolomide in glioblastoma multiforme cells. Tumour Biol..

[73] Li X., Ma W., Wang X., Ci Y., Zhao Y. (2018). Annexin A5 overexpression might suppress proliferation and metastasis of human uterine cervical carcinoma cells. Cancer Biomark..

[74] Sato H., Ogata H., De Luca L.M. (2000). Annexin V inhibits the 12-O-tetradecanoylphorbol-13-acetate-induced activation of Ras/extracellular signal-regulated kinase (ERK) signaling pathway upstream of Shc in MCF-7 cells. Oncogene.

[75] Xue G., Hao L.Q., Ding F.X., Mei Q., Huang J.J., Fu C.G., Yan H.L., Sun S.H. (2009). Expression of annexin A5 is associated with higher tumor stage and poor prognosis in colorectal adenocarcinomas. J. Clin. Gastroenterol..

[76] Mitra A.P., Almal A.A., George B., Fry D.W., Lenehan P.F., Pagliarulo V., Cote R.J., Datar R.H., Worzel W.P. (2006). The use of genetic programming in the analysis of quantitative gene expression profiles for identification of nodal status in bladder cancer. BMC Cancer.

[77] Dooley T.P., Reddy S.P., Wilborn T.W., Davis R.L. (2003). Biomarkers of human cutaneous squamous cell carcinoma from tissues and cell lines identified by DNA microarrays and qRT-PCR. Biochem. Biophys. Res. Commun..

[78] Hawkins T.E., Das D., Young B., Moss S.E. (2002). DT40 cells lacking the Ca2+-binding protein annexin 5 are resistant to Ca2+-dependent apoptosis. Proc. Natl. Acad. Sci. USA.

[79] Luo C., Ji X., Fan J., Hou Z., Wang T., Wu B., Ni C. (2016). Annexin A5 promotes macrophage activation and contributes to pulmonary fibrosis induced by silica particles. Toxicol. Ind. Health.

[80] Zhang Z., Zhang Y., Zhou R. (2020). Loss of Annexin A5 expression attenuates the lipopolysaccharide-induced inflammatory response of rat alveolar macrophages. Cell Biol. Int..

[81] Younossi Z., Anstee Q.M., Marietti M., Hardy T., Henry L., Eslam M., George J., Bugianesi E. (2018). Global burden of NAFLD and NASH: Trends, predictions, risk factors and prevention. Nat. Rev. Gastroenterol. Hepatol..

[82] Rodríguez-Prados J.C., Través P.G., Cuenca J., Rico D., Aragonés J., Martín-Sanz P., Cascante M., Boscá L. (2010). Substrate fate in activated macrophages: A comparison between innate, classic, and alternative activation. J. Immunol..

[83] Xu F., Guo M., Huang W., Feng L., Zhu J., Luo K., Gao J., Zheng B., Kong L.D., Pang T. (2020). Annexin A5 regulates hepatic macrophage polarization via directly targeting PKM2 and ameliorates NASH. Redox Biol..

[84] Tang J., Qin Z., Han P., Wang W., Yang C., Xu Z., Li R., Liu B., Qin C., Wang Z. (2017). High Annexin A5 expression promotes tumor progression and poor prognosis in renal cell carcinoma. Int. J. Oncol..

[85] Lu B., Zhao J., Xu L., Xu Y., Wang X., Peng J. (2012). Identification of molecular target proteins in berberine-treated cervix adenocarcinoma HeLa cells by proteomic and bioinformatic analyses. Phytother. Res..

[86] Guo W.X., Man X.B., Yuan H.X., Shi J., Xue J., Wu M.C., Cheng S.Q. (2007). Proteomic analysis on portal vein tumor thrombus-associated proteins for hepatocellular carcinoma. Zhonghua Yi Xue Za Zhi.

[87] Sofiadis A., Becker S., Hellman U., Hultin-Rosenberg L., Dinets A., Hulchiy M., Zedenius J., Wallin G., Foukakis T., Höög A. (2012). Proteomic profiling of follicular and papillary thyroid tumors. Eur. J. Endocrinol..

[88] Wu X., Tang Y., Huang W. (2011). Identification of proteins interacting with multidrug resistance protein in gastric cancer. World J. Gastroenterol..

[89] Tang S., Huang W., Zhong M., Yin L., Jiang H., Hou S., Gan P., Yuan Y. (2012). Identification Keratin 1 as a cDDP-resistant protein in nasopharyngeal carcinoma cell lines. J. Proteom..

[90] Bedrood S., Jayasinghe S., Sieburth D., Chen M., Erbel S., Butler P.C., Langen R., Ritzel R.A. (2009). Annexin A5 directly interacts with amyloidogenic proteins and reduces their toxicity. Biochemistry.

[91] Bartolome F., Krzyzanowska A., de la Cueva M., Pascual C., Antequera D., Spuch C., Villarejo-Galende A., Rabano A., Fortea J., Alcolea D. (2020). Annexin A5 prevents amyloid-β-induced toxicity in choroid plexus: Implication for Alzheimer’s disease. Sci. Rep..

[92] Sohma H., Imai S.-I., Takei N., Honda H., Matsumoto K., Utsumi K., Matsuki K., Hashimoto E., Saito T., Kokai Y. (2013). Evaluation of annexin A5 as a biomarker for Alzheimer’s disease and dementia with lewy bodies. Front. Aging Neurosci..

[93] Schurgers L.J., Burgmaier M., Ueland T., Schutters K., Aakhus S., Hofstra L., Gullestad L., Aukrust P., Hellmich M., Narula J. (2016). Circulating annexin A5 predicts mortality in patients with heart failure. J. Intern. Med..

[94] Jung Y.H., Han D., Shin S.H., Kim E.-K., Kim H.-S. (2020). Proteomic identification of early urinary-biomarkers of acute kidney injury in preterm infants. Sci. Rep..

[95] Lee G., Pollard H.B., Arispe N. (2002). Annexin 5 and apolipoprotein E2 protect against Alzheimer’s amyloid-beta-peptide cytotoxicity by competitive inhibition at a common phosphatidylserine interaction site. Peptides.

[96] Yamaguchi M., Kokai Y., Imai S.-I., Utsumi K., Matsumoto K., Honda H., Mizue Y., Momma M., Maeda T., Toyomasu S. (2010). Investigation of annexin A5 as a biomarker for Alzheimer’s disease using neuronal cell culture and mouse model. J. Neurosci. Res..

[97] Wu A.H. (2006). Serial testing of B-type natriuretic peptide and NTpro-BNP for monitoring therapy of heart failure: The role of biologic variation in the interpretation of results. Am. Heart J..

[98] Ravassa S., González A., López B., Beaumont J., Querejeta R., Larman M., Díez J. (2007). Upregulation of myocardial Annexin A5 in hypertensive heart disease: Association with systolic dysfunction. Eur. Heart J..

[99] Jakubowska A., Kiliś-Pstrusińska K. (2020). Annexin V in children with idiopathic nephrotic syndrome treated with cyclosporine A. Adv. Clin. Exp. Med..

[100] Lee S.H., Lee P.H., Kim B.G., Hong J., Jang A.S. (2018). Annexin A5 Protein as a Potential Biomarker for the Diagnosis of Asthma. Lung.

[101] Leist M., Jäättelä M. (2001). Triggering of apoptosis by cathepsins. Cell Death Differ..

[102] Reutelingsperger C.P., Dumont E., Thimister P.W., van Genderen H., Kenis H., van de Eijnde S., Heidendal G., Hofstra L. (2002). Visualization of cell death in vivo with the annexin A5 imaging protocol. J. Immunol. Methods.

[103] Hengartner M.O. (2000). The biochemistry of apoptosis. Nature.

[104] Savill J., Fadok V. (2000). Corpse clearance defines the meaning of cell death. Nature.

[105] Keen H.G., Dekker B.A., Disley L., Hastings D., Lyons S., Reader A.J., Ottewell P., Watson A., Zweit J. (2005). Imaging apoptosis in vivo using 124I-annexin V and PET. Nucl. Med. Biol..

[106] Taki J., Higuchi T., Kawashima A., Fukuoka M., Kayano D., Tait J.F., Matsunari I., Nakajima K., Kinuya S., Strauss H.W. (2007). Effect of postconditioning on myocardial 99mTc-annexin-V uptake: Comparison with ischemic preconditioning and caspase inhibitor treatment. J. Nucl. Med..

[107] Yang D.J., Azhdarinia A., Wu P., Yu D.F., Tansey W., Kalimi S.K., Kim E.E., Podoloff D.A. (2001). In vivo and in vitro measurement of apoptosis in breast cancer cells using 99mTc-EC-annexin V. Cancer Biother. Radiopharm..

[108] Grierson J.R., Yagle K.J., Eary J.F., Tait J.F., Gibson D.F., Lewellen B., Link J.M., Krohn K.A. (2004). Production of [F-18]fluoroannexin for imaging apoptosis with PET. Bioconjug. Chem..

[109] Bauwens M., De Saint-Hubert M., Devos E., Deckers N., Reutelingsperger C., Mortelmans L., Himmelreich U., Mottaghy F.M., Verbruggen A. (2011). Site-specific 68Ga-labeled Annexin A5 as a PET imaging agent for apoptosis. Nucl. Med. Biol..

[110] Kurihara H., Yang D.J., Cristofanilli M., Erwin W.D., Yu D.F., Kohanim S., Mendez R., Kim E.E. (2008). Imaging and dosimetry of 99mTc EC annexin V: Preliminary clinical study targeting apoptosis in breast tumors. Appl. Radiat. Isot..

[111] Kenis H., Zandbergen H.R., Hofstra L., Petrov A.D., Dumont E.A., Blankenberg F.D., Haider N., Bitsch N., Gijbels M., Verjans J.W. (2010). Annexin A5 uptake in ischemic myocardium: Demonstration of reversible phosphatidylserine externalization and feasibility of radionuclide imaging. J. Nucl. Med..

[112] Hua Z., Xie D. (2018). Doxorubicin and Annexin v Conjugates and Its Preparation Method and Application: ZL201110217929.6.

[113] Linke B., Abeler-Dörner L., Jahndel V., Kurz A., Mahr A., Pfrang S., Linke L., Krammer P.H., Weyd H. (2015). The tolerogenic function of annexins on apoptotic cells is mediated by the annexin core domain. J. Immunol..

[114] Plato A., Willment J.A., Brown G.D. (2013). C-type lectin-like receptors of the dectin-1 cluster: Ligands and signaling pathways. Int. Rev. Immunol..

[115] Bode K., Bujupi F., Link C., Hein T., Zimmermann S., Peiris D., Jaquet V., Lepenies B., Weyd H., Krammer P.H. (2019). Dectin-1 Binding to Annexins on Apoptotic Cells Induces Peripheral Immune Tolerance via NADPH Oxidase-2. Cell Rep..

[116] Li L., Zou J., Dai Y., Fan W., Niu G., Yang Z., Chen X. (2020). Burst release of encapsulated annexin A5 in tumours boosts cytotoxic T-cell responses by blocking the phagocytosis of apoptotic cells. Nat. Biomed. Eng..

[117] Chang W., Fa H., Xiao D., Wang J. (2020). Targeting phosphatidylserine for Cancer therapy: Prospects and challenges. Theranostics.

[118] Frey B., Schildkopf P., Rödel F., Weiss E.M., Munoz L.E., Herrmann M., Fietkau R., Gaipl U.S. (2009). AnnexinA5 renders dead tumor cells immunogenic--implications for multimodal cancer therapies. J. Immunotoxicol..

[119] Kang T.H., Park J.H., Yang A., Park H.J., Lee S.E., Kim Y.S., Jang G.Y., Farmer E., Lam B., Park Y.M. (2020). Annexin A5 as an immune checkpoint inhibitor and tumor-homing molecule for cancer treatment. Nat. Commun..

[120] Li C., Huang J., Yang G. (2001). A rabbit femoral artery thrombosis model induced by balloon injury. J. Nanjing Med. Univ..

[121] Tait J.F., Engelhardt S., Smith C., Fujikawa K. (1995). Prourokinase-annexin V chimeras. Construction, expression, and characterization of recombinant proteins. J. Biol. Chem..

[122] Tanaka K., Einaga K., Tsuchiyama H., Tait J.F., Fujikawa K. (1996). Preparation and characterization of a disulfide-linked bioconjugate of annexin V with the B-chain of urokinase: An improved fibrinolytic agent targeted to phospholipid-containing thrombi. Biochemistry.

[123] Huang X., Ding W.Q., Vaught J.L., Wolf R.F., Morrissey J.H., Harrison R.G., Lind S.E. (2006). A soluble tissue factor-annexin V chimeric protein has both procoagulant and anticoagulant properties. Blood.

[124] Kuypers F.A., Larkin S.K., Emeis J.J., Allison A.C. (2007). Interaction of an annexin V homodimer (Diannexin) with phosphatidylserine on cell surfaces and consequent antithrombotic activity. Thromb. Haemost..

[125] Jing J., Sun Y. (2019). An α(IIb)β(3)- and phosphatidylserine (PS)-binding recombinant fusion protein promotes PS-dependent anticoagulation and integrin-dependent antithrombosis. J. Biol. Chem..

[126] Pan Y., Ren X., Wang S., Li X., Luo X., Yin Z. (2017). Annexin V-Conjugated Mixed Micelles as a Potential Drug Delivery System for Targeted Thrombolysis. Biomacromolecules.

[127] Subramanian S., Ugoya S.O., Zhao Z., McRobb L.S., Grau G.E., Combes V., Inglis D.W., Gauden A.J., Lee V.S., Moutrie V. (2018). Stable thrombus formation on irradiated microvascular endothelial cells under pulsatile flow: Pre-testing annexin V-thrombin conjugate for treatment of brain arteriovenous malformations. Thromb. Res..

[128] Espinosa G., Cervera R., Font J., Shoenfeld Y. (2003). Antiphospholipid syndrome: Pathogenic mechanisms. Autoimmun. Rev..

[129] Chighizola C.B., Ubiali T., Meroni P.L. (2015). Treatment of thrombotic antiphospholipid syndrome: The rationale of current management-an insight into future approaches. J. Immunol. Res..

[130] Wu X.X., Guller S., Rand J.H. (2011). Hydroxychloroquine reduces binding of antiphospholipid antibodies to syncytiotrophoblasts and restores annexin A5 expression. Am. J. Obstet. Gynecol..

[131] Eggleston P.A., Kagey-Sobotka A., Lichtenstein L.M. (1987). A comparison of the osmotic activation of basophils and human lung mast cells. Am. Rev. Respir. Dis..

[132] Tahan F., Akar H.H., Saraymen B. (2015). Exhaled breath condensate annexin A5 levels in exercise-induced bronchoconstriction in asthma: A preliminary study. Allergol. Immunopathol..

[133] Garnier Y., Ferdinand S., Garnier M., Cita K.C., Hierso R., Claes A., Connes P., Hardy-Dessources M.D., Lapouméroulie C., Lemonne N. (2020). Plasma microparticles of sickle patients during crisis or taking hydroxyurea modify endothelium inflammatory properties. Blood.

[134] Zahran A.M., El-Badawy O., Ali W.A., Mahran Z.G., Mahran E., Rayan A. (2021). Circulating microparticles and activated platelets as novel prognostic biomarkers in COVID-19; relation to cancer. PLoS ONE.

[135] Garnier Y., Claude L., Hermand P., Sachou E., Claes A., Desplan K., Chahim B., Roger P.M., Martino F., Colin Y. (2022). Plasma microparticles of intubated COVID-19 patients cause endothelial cell death, neutrophil adhesion and netosis, in a phosphatidylserine-dependent manner. Br. J. Haematol..

